# Screening of Drugs Inhibiting *In vitro* Oligomerization of Cu/Zn-Superoxide Dismutase with a Mutation Causing Amyotrophic Lateral Sclerosis

**DOI:** 10.3389/fmolb.2016.00040

**Published:** 2016-08-09

**Authors:** Itsuki Anzai, Keisuke Toichi, Eiichi Tokuda, Atsushi Mukaiyama, Shuji Akiyama, Yoshiaki Furukawa

**Affiliations:** ^1^Laboratory for Mechanistic Chemistry of Biomolecules, Department of Chemistry, Keio UniversityYokohama, Japan; ^2^Research Center of Integrative Molecular Systems, Institute for Molecular ScienceOkazaki, Japan; ^3^Department of Functional Molecular Science, SOKENDAI (The Graduate University for Advanced Studies)Okazaki, Japan

**Keywords:** Cu/Zn-superoxide dismutase, amyotrophic lateral sclerosis, protein misfolding, protein aggregation, drug screening

## Abstract

Dominant mutations in Cu/Zn-superoxide dismutase (*SOD1*) gene have been shown to cause a familial form of amyotrophic lateral sclerosis (*SOD1*-ALS). A major pathological hallmark of this disease is abnormal accumulation of mutant SOD1 oligomers in the affected spinal motor neurons. While no effective therapeutics for *SOD1*-ALS is currently available, SOD1 oligomerization will be a good target for developing cures of this disease. Recently, we have reproduced the formation of SOD1 oligomers abnormally cross-linked *via* disulfide bonds in a test tube. Using our *in vitro* model of SOD1 oligomerization, therefore, we screened 640 FDA-approved drugs for inhibiting the oligomerization of SOD1 proteins, and three effective classes of chemical compounds were identified. Those hit compounds will provide valuable information on the chemical structures for developing a novel drug candidate suppressing the abnormal oligomerization of mutant SOD1 and possibly curing the disease.

## Introduction

Amyotrophic lateral sclerosis (ALS) is a fatal neurodegenerative disease characterized by the progressive loss of motor neurons, leading to muscle atrophy and total paralysis. While increasing numbers of causative genes for a familial form of ALS have been identified, ~90% of the ALS cases are sporadic, and the pathomechanism still remains largely unknown (Andersen and Al-Chalabi, 2011; Renton et al., 2014). Also, no clinically approved drug is available for ALS except Riluzole, which can extend the median survival by only a few months (Genc and Ozdinler, 2014); therefore, a great need for new ALS therapeutics is obvious. Despite this, it remains obscure what triggers sporadic ALS, hampering the identification of molecular target(s) for the development of drugs to ameliorate and eventually prevent this devastating disease.

As mentioned, several genes responsible for a familial form of ALS cases have been identified (Abel et al., 2012), among which mutations in Cu/Zn-superoxide dismutase (*SOD1*) gene are the most common cause (about 20%) of familial ALS cases (*SOD1*-ALS; Rosen et al., 1993). A pathological hallmark observed in *SOD1*-ALS cases is abnormal accumulation of mutant SOD1 proteins in the affected motor neurons as inclusions (Bruijn et al., 1998). Furthermore, *in vivo* as well as *in vitro* experiments have shown that pathogenic mutations trigger the misfolding of SOD1 proteins and then lead to the formation of insoluble SOD1 oligomers/aggregates (reviewed in Furukawa, 2012). Misfolding of SOD1 proteins is thus one of the promising targets to develop therapeutics of *SOD1*-ALS cases.

SOD1 is an antioxidant enzyme detoxifying superoxide radicals and becomes enzymatically active by binding copper and zinc ions and forming a highly conserved intramolecular disulfide bond (McCord and Fridovich, 1969; Furukawa et al., 2004). Given that these post-translational processes are known to stabilize the native conformation of SOD1 proteins, pathogenic mutations are considered to decrease the conformational stability of SOD1 by compromising binding of the metal ions and/or formation of the disulfide bond (Furukawa and O'Halloran, 2005; Rodriguez et al., 2005). Regardless of the presence or absence of metal binding and/or disulfide formation, furthermore, most of pathogenic mutations are shown to decrease structural stability of SOD1 proteins (Furukawa and O'Halloran, 2005; Rodriguez et al., 2005); therefore, the decrease in the structural stability of SOD1 is considered to trigger the misfolding into a toxic conformer(s). It is thus well expected that misfolding of SOD1 into oligomers and aggregates can be suppressed by stabilizing the native conformation of SOD1.

A native form of SOD1 is a homodimer, and the mutation-induced monomerization of SOD1 has been suggested to trigger the aggregation (Rakhit et al., 2004). Actually, introduction of a disulfide bond between the SOD1 subunits by either genetic engineering (Ray et al., 2004) or chemical cross-linkers (Auclair et al., 2010) has been shown to forcedly stabilize the SOD1 dimeric structure. The coordination of a metallo-complex, cisplatin, at the dimer interface was also shown to be effective to suppressing the SOD1 oligomerization in the cultured cells as well as in a test tube (Banci et al., 2012). To more reversibly stabilize the natively dimeric SOD1 conformation, small drugs that can bind at the dimer interface were identified by extensive *in silico* screening of ~1.5 million compounds (Ray et al., 2005; Nowak et al., 2010). These drugs were found to protect A4V-mutant SOD1 from chemical unfolding induced by guanidine hydrochloride (Gdn) and also significantly slow the aggregation (Ray et al., 2005; Nowak et al., 2010). The binding site of those drugs in SOD1, however, needs to be experimentally confirmed in more detail; actually, some of the drugs were shown to bind at the alternative site other than the dimer interface (Antonyuk et al., 2010; Wright et al., 2013). Further studies will also be required to confirm the reproducibility on the stabilizing effects of those drugs on SOD1 (Wright et al., 2013), and *in vivo* efficacy of those dimer-stabilizing drugs remains to be tested. To identify drugs that can reduce the intracellular aggregation of mutant SOD1, moreover, high throughput screening of ~50,000 small molecules has been performed in the cultured cells expressing mutant SOD1 fused with a fluorescent protein, GFP (Benmohamed et al., 2011). Aggregation properties of SOD1 proteins have, however, been reported to be affected by the fusion with a GFP tag (Stevens et al., 2010). While efforts have been made to stabilize mutant SOD1 in a drug-based approach, no drugs are promising for animal and clinical trials, and we hence still need to explore and test more numbers of potent inhibitors for aggregation/oligomerization of mutant SOD1 proteins.

Here, we have performed screening of FDA-approved drugs in our experimental setup reproducing SOD1 oligomerization *in vitro* and identified drugs that can suppress the formation of insoluble SOD1 oligomers. Previously, we have shown that pathogenic SOD1 proteins form SDS-resistant oligomers crosslinked *via* disulfide bonds in the spinal cords of symptomatic ALS-model mice expressing mutant SOD1 (Furukawa et al., 2006). We also recently reported that formation of disulfide-crosslinked oligomers was reproduced in a test tube by incubation of metal-deficient (apo) SOD1 with the disulfide bond (apo-SOD1^S-S^) in the presence of a denaturant (Toichi et al., 2013). The hit compounds in a library of FDA-approved drugs efficiently suppressed the formation of insoluble SOD1 oligomers crosslinked *via* disulfide bonds. The structural/chemical information on those compounds will hence be useful to design a pharmaceutical drug for *SOD1*-ALS with aggregation-inhibiting properties.

## Materials and methods

### Preparation of proteins

Expression and purification of SOD1 with G37R mutation were performed as described previously (Furukawa et al., 2016). Briefly, SOD1(G37R) was expressed in *E. coli* SHuffle™ (New England BioLabs) as a fusion protein with an N-terminal 6x His tag and was purified with Ni^2+^-affinity chromatography using HisTrap HP column (1 mL, GE Healthcare). The purified proteins were dialyzed against 50 mM sodium acetate, 100 mM NaCl, and 10 mM EDTA at pH 4.0, which removed metal ions bound to the His-tagged SOD1(G37R). After proteolytic cleavage of the His tag with thrombin, the demetallated and untagged SOD1(G37R) was further purified with gel-filtration chromatography using Cosmosil 5Diol-300-II column (nacalai tesque). Presence of the intramolecular disulfide bond in SOD1 was confirmed based upon its distinct electrophoretic mobility in non-reducing SDS-PAGE (Toichi et al., 2013). HTT^EX1^(42Q) were prepared as described previously (Mitomi et al., 2012). Concentrations of SOD1 and HTT^EX1^(42Q) proteins were spectroscopically determined from the absorbance at 280 nm in the presence of 6 M guanidine hydrochloride (Gdn) by using 5625 and 42,860 cm^−1^M^−1^ as an extinction coefficient, respectively.

### Screening of drugs inhibiting the SOD1 oligomerization

To examine effects of drugs on the abnormal oligomerization of SOD1 *in vitro*, apo-SOD1(G37R)^S-S^ (20 μM, 150 μL) in a buffer at pH 7.4 containing 100 mM Na-Pi, 100 mM NaCl and 5 mM EDTA (NNE buffer) with 1 M Gdn was first prepared in each well of a 96-well-plate. Then, 1.5 μL of a 2.0 g/L stock solution of drugs in DMSO was added to the protein sample solution in a well of the plate (the final concentration of drugs tested was 20 μg/mL). In this study, we have examined 640 drugs in the FDA approved drug library (#BML-2841J-0100, Japanese version, Enzo Life Science). The oligomerization reaction was started by shaking the samples with a POM ball (3/32 inch, SANPLATEC) in a plate shaker (MBR-022UP, TAITEC) at 1200 rpm, 37°C and monitored by the increase of solution turbidity (absorbance increase at 350 nm). After 12 h of agitation, the samples were collected and ultracentrifuged at 110,000 × *g* for 15 min. to obtain soluble supernatant and insoluble pellet separately. After removing Gdn in the soluble supernatant with PAGE Clean Up Kit (nacalai tesque), the proteins in both fractions were analyzed by non-reducing SDS-PAGE.

### Electrophoresis

To protect free thiols from aberrant oxidation during electrophoresis, proteins were reacted with a thiol-specific modifier, iodoacetamide (IA); the samples were first dissolved in a buffer at pH 8.0 containing 100 mM Na-Pi, 2% SDS, and 100 mM IA and then incubated at 37°C for an hour. Followed by the addition of an SDS-PAGE sample buffer without any reductants, the protein samples were boiled at 100°C for 5 min. and then loaded on a 12.5% resolving polyacrylamide gel with a 5% stacking gel. After electrophoresis, the protein bands on a gel were visualized by Coomassie Brilliant Blue.

### Differential scanning calorimetry

Effects of drugs on the thermal stability of SOD1 were examined by differential scanning calorimetry (DSC) using MicroCal VP-DSC (GE Healthcare). Apo-SOD1(G37R)^S-S^ (0.3 g/L; *ca*. 20 μM) in the NNE buffer with or without a drug (20 μg/mL) was first degassed using ThermoVac (GE Healthcare) at 20°C for 5 min., and then the thermograms were obtained by increasing temperature from 15° to 75°C at a scan rate of 1.0°C/min. Baselines were collected by using the NNE buffer containing corresponding drugs and DMSO. The thermograms of protein samples were corrected with the baselines and then normalized by protein concentrations.

### MALDI-TOF mass spectrometry

To map the disulfide bond in SOD1, the limited proteolysis followed by the peptide mapping was performed. As described, apo-SOD1(G37R)^S-S^ was agitated in the presence of 1 M Gdn for 2 h at 37°C, and a soluble fraction of the sample was collected by ultracentrifugation at 110,000 × *g* for 15 min. Proteins in the soluble fraction were first precipitated using PAGE Clean Up Kit and then redissolved in the NNE buffer containing 100 mM IA and 6 M Gdn. After incubation at 37°C for an hour, by which free thiolate groups were alkylated and thus protected, the buffer was exchanged to 100 mM Na-Pi at pH 8.0 by using a Zeba™ Spin Desalting Column (Thermo). SOD1 proteins (48 μg) collected by the spin column were treated with 0.24 μg of porcine pancreas trypsin in a sequencing grade (Wako) and incubated at 37°C for 16 h. After addition of 0.1% trifluoroacetic acid and 6 M Gdn, the trypsinized samples were desalted with ZipTip C18 (Millipore) and then spotted on a MALDI target plate with human ACTH (18–39) (*m*/*z*, 2466.6; Wako) and bovine pancreas insulin (*m*/*z*, 5734.5; nacalai tesque) as internal standards and with α-cyano-4-hydroxycinnamic acid (Wako) as a matrix. Spectra were obtained using Ultraflex™ (Bruker Daltonics) in linear mode, and mass peaks were assigned using MS Bridge (University of California, San Francisco, CA).

## Results and discussion

### Oligomerization of mutant SOD1 in the destabilizing conditions

**Figure 1:**
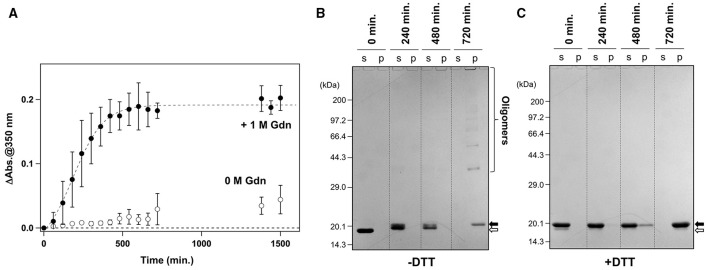
**Formation of insoluble apo-SOD1(G37R)^S-S^ oligomers by structural destabilization. (A)** Formation kinetics of insoluble aggregates from 20 μM apo-SOD1(G37R)^S-S^ was monitored by the increase of solution turbidity (absorbance at 350 nm) in the absence (open circles) and presence (filled circles) of 1 M Gdn. Experiments were repeated more than three times to estimate error bars (standard deviation). **(B,C)** During the aggregation of apo-SOD1(G37R)^S-S^ shown in **(A)**, aliquots were taken from the sample solution at the indicated time, fractionated into soluble supernatant (s), and insoluble pellet (p) by ultracentrifugation and then analyzed by **(B)** non-reducing SDS-PAGE and **(C)** reducing SDS-PAGE after treatment of the samples with DTT. The bands corresponding to SOD1 proteins with and without the canonical disulfide bond are indicated by open and filled arrows, respectively.

It has been reported that agitation of apo-SOD1^S-S^ forms insoluble aggregates in the presence of 1 M Gdn (Chattopadhyay et al., 2008; Toichi et al., 2013). As shown in Figure 1A, we confirmed the increase of solution turbidity by agitation of 20 μM apo-SOD1^S-S^ with a pathogenic mutation, G37R [apo-SOD1(G37R)^S-S^], at 37°C, indicating the formation of insoluble aggregates. In contrast, the agitation of apo-SOD1(G37R)^S-S^ in the absence of Gdn did not result in the formation of insoluble aggregates (Figure 1A). To further confirm the insolubilization of apo-SOD1(G37R)^S-S^ by its agitation in the presence of 1 M Gdn at 37°C, the samples were fractionated into soluble supernatant and insoluble pellets by ultracentrifugation and analyzed by non-reducing SDS-PAGE (Figure 1B). Increase in the solution turbidity was confirmed to associate with the formation of insoluble SOD1 oligomers that were sensitive to the reducing reagent, dithiothreitol (DTT; Figure 1C). Namely, in the current reaction conditions, apo-SOD1(G37R)^S-S^ was converted to the insoluble SOD1 oligomers crosslinked *via* disulfide bonds (S-S oligomers).

We also noted that, before the formation of S-S oligomers, the electrophoretic mobility of SOD1 under the denatured condition (i.e., in SDS-PAGE) became slightly retarded and blurred (compare the bands in the soluble fractions at 240 vs. 0 min. in Figure 1B). Treatment of the samples with DTT again collapsed those distinct SOD1 bands into a single band corresponding to the disulfide-reduced SOD1 (SOD1^SH^; Figure 1C). Consistent with our previous findings (Toichi et al., 2013), these results indicate that the disulfide shuffling occurs first within a SOD1 molecule and then between the molecules to form the insoluble S-S oligomers. Given that accumulation of the insoluble S-S oligomers is known as a pathological hallmark observed in the spinal cords of the affected mice expressing mutant human SOD1 proteins (Deng et al., 2006; Furukawa et al., 2006), we attempted to seek small molecules that can inhibit the formation of insoluble S-S oligomers.

### Screening of drugs for the activity to suppress the oligomerization of mutant SOD1

**Figure 2:**
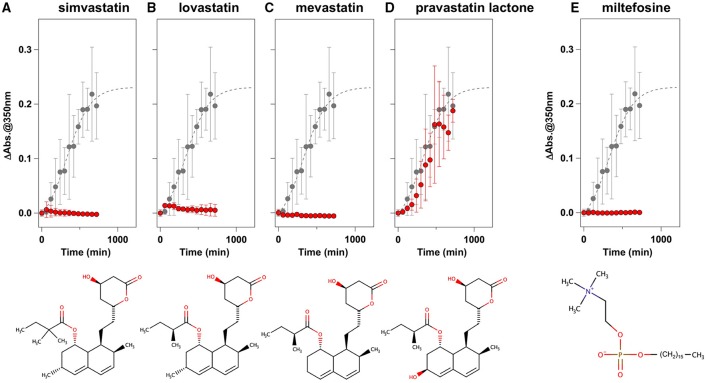
**Statins and miltefosine as potent inhibitors for the formation of insoluble apo-SOD1(G37R)^S-S^ aggregates. (A–E)** The turbidity increase of 20 μM apo-SOD1(G37R)^S-S^ in the presence of 1 M Gdn is shown in the presence of the following compounds (red filled circles): **(A)** simvastatin, **(B)** lovastatin, **(C)** mevastatin, **(D)** pravastatin lactone, and **(E)** miltefosine. In each panel, the aggregation kinetics without any drugs was shown as gray filled circles. The kinetics was monitored by changes in the solution turbidity (the absorbance at 350 nm), and the final concentration of drugs added was 20 μg/mL. Experiments were repeated more than three times to estimate error bars (standard deviation). Structures of the chemical compounds were also shown below the panels.

**Figure 3:**
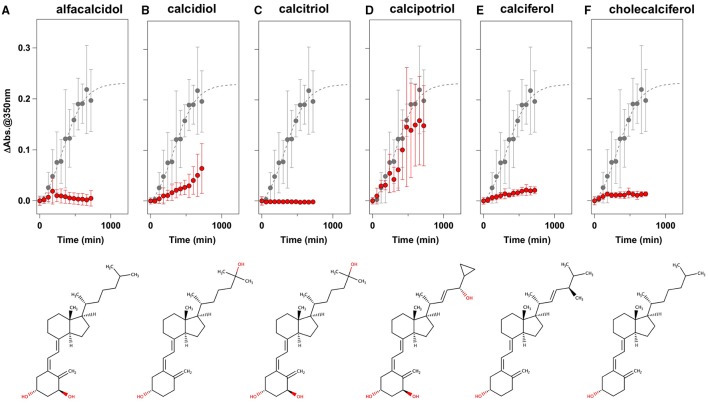
**Vitamin D derivatives as potent inhibitors for the formation of insoluble apo-SOD1(G37R)^S-S^ aggregates. (A–F)** The turbidity increase of 20 μM apo-SOD1(G37R)^S-S^ in the presence of 1 M Gdn is shown in the presence of the following compounds (red filled circles): **(A)** alfacalcidol, **(B)** calcidol, **(C)** calcitriol, **(D)** calcipotriol, **(E)** calciferol, and **(F)** cholecalciferol. In each panel, the aggregation kinetics without any drugs was shown as gray filled circles. The kinetics was monitored by changes in the solution turbidity (the absorbance at 350 nm), and the final concentration of drugs added was 20 μg/mL. Experiments were repeated more than three times to estimate error bars (standard deviation). Structures of the chemical compounds were also shown below the panels.

To facilitate the transfer of possible drug candidates to the clinical trials, we chose a library of 640 drugs, which have been already approved by the U.S. Food and Drug Administration (FDA). Twenty micrometer of apo-SOD1(G37R)^S-S^ in the NNE buffer containing 1 M Gdn were mixed with a drug in the final concentration of 20 μg/mL and shaken at 1200 rpm, 37°C. While, in the absence of any drugs, the solution turbidity (absorbance at 350 nm) of apo-SOD1(G37R)^S-S^ was around 0.2 at 500 min after agitation (Figure 1A), drugs showing the turbidity of < 0.02 at 500 min after agitation were chosen in this study as “hit” compounds probably suppressing the formation of insoluble SOD1 aggregates. In the library of FDA-approved drugs, six compounds were found to almost completely suppress the increase of solution turbidities: simvastatin (Figure 2A), lovastatin (Figure 2B), mevastatin (Figure 2C), miltefosine (Figure 2E), alfacalcidol (Figure 3A), calcidiol (Figure 3B), and calcitriol (Figure 3C). Inhibitory effects of these drugs on the formation of insoluble aggregates were also confirmed in the final concentration of 10 μg/mL (data not shown). Based upon the chemical structure, those hit compounds can be categorized into three groups as follows.

**Figure 4:**
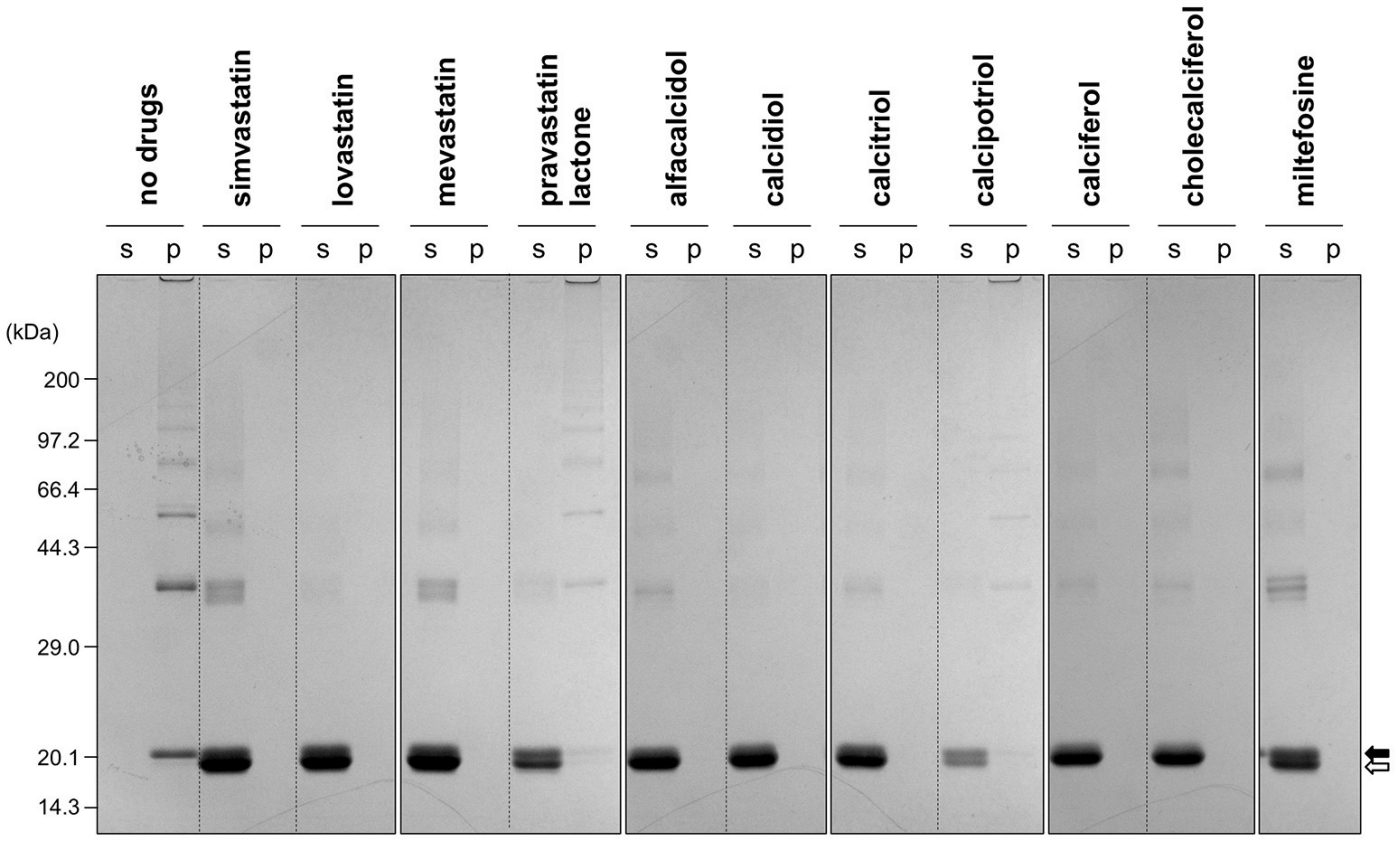
**Formation of insoluble SOD1 oligomers was efficiently suppressed by the hit compounds**. As shown in Figures 2, 3, 20 μM apo-SOD1(G37R)^S-S^ was shaken at 37°C for 12 h in the NNE buffer containing 1 M Gdn in the presence of the indicated drugs (20 μg/mL) and then fractionated into soluble supernatant (s) and insoluble pellet (p) by ultracentrifugation. Both fractions were then analyzed by non-reducing SDS-PAGE using a 12.5% polyacrylamide gel. The position of the band corresponding to SOD1 with and without the canonical disulfide bond was indicated by open and filled arrows, respectively.

The first group contains simvastatin, lovastatin, and mevastatin, all of which are cholesterol-lowering agents by inhibiting the rate-limiting enzyme, HMG-CoA reductase, in cholesterol biosynthesis with quite similar chemical structures composed of hexahydronaphthalene, heptanoic acid lactone, and butanoate groups (Figures 2A–C). In the absence of any drugs, apo-SOD1(G37R)^S-S^ formed insoluble S-S oligomers with no SOD1 proteins in the soluble fraction (Figure 4, no drugs), resulting in the increase of solution turbidity. By addition of those three statins, all SOD1 proteins remained soluble (Figure 4), corroborating the suppression of turbidity increase. Notably, the library of FDA-approved drugs tested in this study contains another statin with the similar structure, pravastatin lactone, which was, however, found to have no effects on the turbidity increase of the apo-SOD1(G37R)^S-S^ sample (Figure 2D) and show reduced ability to suppress the formation of insoluble S-S oligomers (Figure 4). Other statins in the library (nystatin, cilastatin, cerivastatin, and fluvastatin), which have quite distinct chemical structures from e.g., simvastatin, were also tested but exhibited no effects on the SOD1 aggregation *in vitro* (data not shown). Further investigation will be required to describe *in vitro* efficacy of those statins as an inhibitor for SOD1 aggregation, but lovastatin and pravastatin lactone differ in only one structural respect: a substituent in the hexahydronaphthalene group is a methyl group in lovastatin but a hydroxyl group in pravastatin lactone (Figures 2B,D). Hydrophobicity in the hexahydronaphthalene group would hence be necessary for inhibition of the SOD1 oligomerization (*vide infra*).

The second group includes alfacalcidol, calcidiol, and calcitriol (Figures 3A–C), all of which are derivatives of vitamin D. Actually, calciferol (vitamin D_2_) and cholecalciferol (vitamin D_3_) were not included in the FDA-approved drug library used in this study but effectively suppressed the increase of the solution turbidity in the apo-SOD1(G37R)^S-S^ sample (Figures 3E,F). The drug library in this study has another vitamin D derivative, calcipotriol, which was, however, ineffective to the suppression of the solution turbidity increase in the apo-SOD1(G37R)^S-S^ sample (Figure 3D). Furthermore, SOD1 proteins were confirmed to remain soluble without any insoluble SOD1 aggregates/oligomers by adding either one of vitamin D derivatives examined here except calcipotriol (Figure 4). While no obvious correlation was confirmed between the chemical structures and the efficacy to suppress the formation of insoluble SOD1 oligomers, it is interesting to note that the water solubility of calcipotriol is presumably higher than those of the other vitamin D derivatives. More precisely, as summarized in Table 1, the water solubility of the vitamin D derivatives that are effective to the inhibition of SOD1 aggregation ranges from 0.00038 g/L (1.0 μM, cholecalciferol) to 0.0067 g/L (16 μM, calcitriol); in contrast, calcipotriol is expected to have 0.014 g/L (33 μM) of the water solubility. Actually, the water solubility of pravastatin lactone (0.024 g/L, 59 μM), which was unable to suppress the turbidity increase (Figure 2D), is comparable to that of calcipotriol and is significantly higher than those of the other statins that can inhibit the SOD1 aggregation (0.00077–0.0048 g/L; Table 1). Collectively, therefore, the hydrophobicity of the drugs would be an important factor to modulate the efficacy for inhibiting the oligomerization of SOD1. We further speculate that apo-SOD1(G37R)^S-S^ exposes its hydrophobic interior upon structural destabilization and thereby forms abnormal oligomers. The drugs with significant hydrophobicity might hence inhibit such abnormal oligomerization by interacting with the hydrophobic interior of SOD1.

**Table 1:** **Expected values for water solubility of drugs examined in this study**.

**Drugs**	**M.W.^a^**	**Water solubility**		**Data source**
		**g/L**	**μM**	
Simvastatin	418.6	7.7 × 10^−4^	1.8 × 10^0^	ChemIDplus^b^
Lovastatin	404.5	2.1 × 10^−3^	5.3 × 10^0^	ChemIDplus^b^
Mevastatin	390.5	4.8 × 10^−3^	1.2 × 10^1^	ChemIDplus^b^
Pravastatin lactone	406.5	2.4 × 10^−2^	5.9 × 10^1^	ChemIDplus^b^
Alfacalcidol	400.6	1.6 × 10^−3^	4.1 × 10^0^	DrugBank^c^
Calcidol	400.6	2.2 × 10^−3^	5.5 × 10^0^	DrugBank^c^
Calcitriol	416.6	6.7 × 10^−3^	1.6 × 10^1^	DrugBank^c^
Calcipotriol	412.6	1.4 × 10^−2^	3.3 × 10^1^	DrugBank^c^
Calciferol	396.7	4.3 × 10^−4^	1.1 × 10^0^	DrugBank^c^
Cholecalciferol	384.6	3.8 × 10^−4^	1.0 × 10^0^	DrugBank^c^
Miltefosine	407.6	2.2 × 10^−4^	5.0 × 10^−1^	DrugBank^c^
		2.5 × 10^0^	6.1 × 10^3^	Data sheet^d^

a *Molecular weight*.

b *http://chem.sis.nlm.nih.gov/chemidplus/*.

c *http://www.drugbank.ca*.

d *https://www.caymanchem.com/pdfs/63280.pdf*.

Miltefosine, a phospholipid antimicrobial drug, belongs to the last group of our hit compounds (Figure 2E). In the presence of miltefosine, the turbidity increase of apo-SOD1(G37R)^S-S^ was almost completely suppressed (Figure 2E), and no insoluble SOD1 oligomers were detected (Figure 4). The water solubility of miltefosine has been calculated as a very limited value (0.00022 g/L, 0.5 μM, Table 1). Actually, however, miltefosine (Cayman Chemical No. 63280) was soluble in water (2.5 g/L) probably by forming micelles because of its hydrophobic alkane chain with hydrophilic phosphate and amine groups. We thus suppose that the hydrophobic part of miltefosine plays a role in the interaction with misfolded SOD1 and the inhibition of abnormal oligomerization.

### Specificity of the drugs to suppress protein aggregation/oligomerization

Not all drugs with significant hydrophobicity (or limited water solubility) were potent against inhibition of SOD1 oligomerization; for example, cerivastatin and fluvastatin have comparable water solubility (*ca*. 4 × 10^−3^ g/L) to that of simvastatin and mevastatin but did not affect the aggregation kinetics of apo-SOD1(G37R)^S-S^ (data not shown). Accordingly, the chemical structures of our hit compounds will have specific interactions with apo-SOD1(G37R)^S-S^ to inhibit the formation of insoluble oligomers.

**Figure 5:**
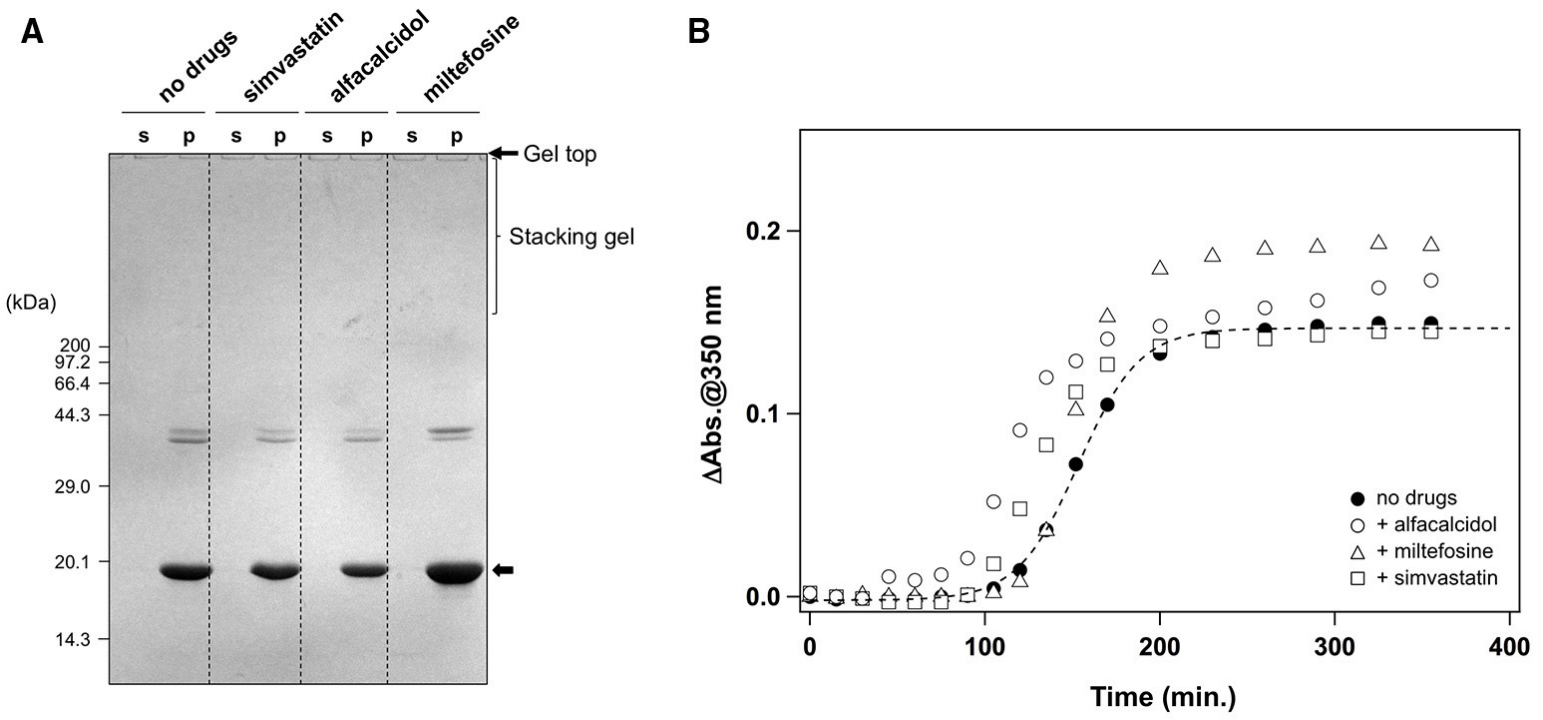
**Specificity of the hit compounds for the inhibition of apo-SOD1(G37R)^S-S^ oligomerization. (A)** 20 μM apo-SOD1(G37R)^SH^ in the NNE buffer with 5 mM DTT was shaken at 1200 rpm at 37°C in the presence or absence of the indicated drugs (20 μg/mL). After 24 h of agitation, the samples were fractionated to soluble supernatant (s) and insoluble pellets (p) by ultracentrifugation and then analyzed by non-reducing SDS-PAGE using a 12.5% polyacrylamide gel. **(B)** Effects of the drugs (20 μg/mL) on the aggregation kinetics of 20 μM HTT^EX1^(42Q) were monitored by solution turbidity (absorbance at 350 nm): no drugs (filled circles), alfacalcidol (open circles), miltefosine (open triangles), and simvastatin (open squares).

In addition to the formation of S-S oligomers, we have previously shown that reduction of the disulfide bond in apo-SOD1 triggers the formation of insoluble aggregates with amyloid-like fibrillar morphologies (Furukawa et al., 2008). As shown in Figure 5A (no drugs), the disulfide-reduced form of apo-SOD1 with G37R mutation [apo-SOD1(G37R)^SH^] was confirmed to form insoluble aggregates by shaking at 37°C overnight. We have then chosen simvastatin, alfacalcidol, and miltefosine as representatives of the three groups mentioned above and tested their efficacy to suppress the formation of amyloid-like insoluble aggregates derived from apo-SOD1(G37R)^SH^. Almost all fractions of apo-SOD1(G37R)^SH^, however, became insoluble even in the presence of those three representative drugs (Figure 5A). Furthermore, in order to test if inhibitory effects of those drugs on the protein aggregation are limited to SOD1, we examined the aggregation of huntingtin with an elongated polyQ tract, which is a pathological hallmark of Huntington disease. As shown in Figure 5B, the translation product of exon 1 in the huntingtin gene with 42 consecutive glutamines [HTT^EX1^(42Q)] formed insoluble aggregates in a sigmoidal kinetics; however, no inhibitory effects of the drugs on the aggregation of HTT^EX1^(42Q) were confirmed. Furthermore, the three representative drugs inhibited the turbidity increase in the aggregation of apo-SOD1^S-S^ with the other ALS-causing mutation, G85R (data not shown). These results thus show that the drugs (simvastatin, alfacalcidol, and miltefosine) can effectively and also specifically suppress the formation of insoluble S-S oligomers derived from apo-SOD1^S-S^ proteins. Nonetheless, it remains obscure how those drugs interact with SOD1 to keep it soluble.

### The drugs inhibit the interactions between misfolded SOD1 molecules leading to formation of insoluble aggregates

We first supposed that the drugs might prevent apo-SOD1(G37R)^S-S^ from aggregation/oligomerization by stabilizing its folded conformation, and we thus examined effects of the drugs on the thermostability of apo-SOD1(G37R)^S-S^ by using differential scanning calorimetry (DSC). The melting temperature, *T*_m_, of apo-SOD1(G37R)^S-S^ was, however, unaffected by the drugs: 42.2°C in the absence of any drugs; 41.6, 41.9, and 42.2°C in the presence of simvastatin, alfacalcidol, and miltefosine, respectively. It is thus unlikely that the drugs interact with the folded apo-SOD1(G37R)^S-S^ to stabilize it.

**Figure 6:**
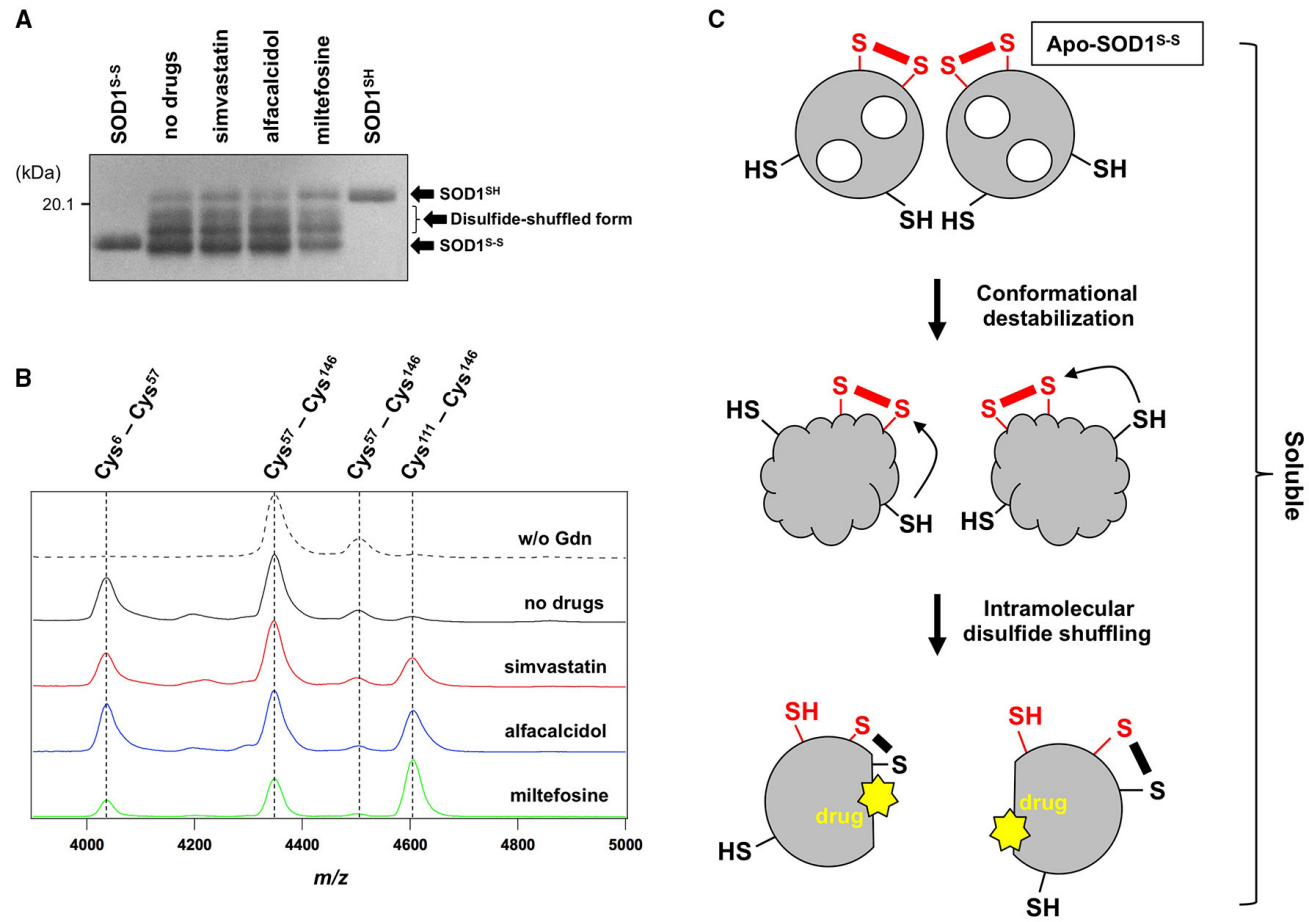
**The drugs could not inhibit the disulfide shuffling within an apo-SOD1(G37R)^S-S^ molecule. (A)** Apo-SOD1(G37R)^S-S^ (20 μM) was agitated for 2 h in the presence of 1 M Gdn with and without the indicated drugs, and the soluble fraction obtained with ultracentrifugation was analyzed by non-reducing SDS-PAGE using a 12.5% polyacrylamide gel in the presence of 5 M urea. As controls, SOD1^SH^ and SOD1^S-S^ were also loaded on a gel. **(B)** After agitated for 2 h at 37°C in the presence of 1 M Gdn, the soluble fractions of apo-SOD1(G37R)^S-S^ were trypsinized and analyzed by MALDI-TOF mass spectrometry (see Materials and methods). Reductant-sensitive mass peaks observed at *m*/*z* 4038, 4350, 4507, and 4607 were assigned to the peptides containing a disulfide bond between Cys 6 and Cys57, Cys 57 and Cys 146, Cys 57 and Cys 146, and Cys 111 and Cys 146, respectively. **(C)** Schematic representation of the SOD1 aggregation pathway inhibited by the drugs. Conformational destabilization of apo-SOD1^S-S^ triggers the intramolecular shuffling of the disulfide bond, and the drugs in this study are considered to interact with the disulfide-shuffled conformation of SOD1 and suppress the interaction of those disulfide-shuffled SOD1 proteins that leads to the formation of insoluble aggregates.

While the drugs completely suppressed the formation of insoluble S-S oligomers, we noted that distinct, albeit slight, amounts of disulfide cross-linked oligomers were observed also in the soluble fraction in the presence of the drugs (Figure 4). Furthermore, the SOD1 band around 20 kDa was blurred in the presence of all drugs tested (indicated by arrows in Figure 4), suggesting the disulfide shuffling within a SOD1 molecule (Figure 1B). Therefore, the drugs might not act on the folded conformation of apo-SOD1^S-S^ so as to inhibit the disulfide shuffling; rather, the drugs would prevent aberrant interactions among the disulfide-shuffled SOD1s that possibly lead to the formation of insoluble aggregates. In order to resolve those blurred bands, the soluble fractions of apo-SOD1^S-S^ shaken at 1200 rpm in the presence and absence of the drugs for 2 h were examined with SDS-PAGE using a polyacrylamide gel containing 5 M urea. As shown in Figure 6A, multiple bands with distinct electrophoretic mobilities (at least four major bands) became apparent after agitation of apo-SOD1(G37R)^S-S^ regardless of the presence or absence of the drugs. By treatment of the samples with the reducing agent, DTT, the multiple bands were collapsed into the single band corresponding to that of SOD1^SH^ (data not shown), corroborating that the multiplicity of bands in Figure 6A is caused by the intramolecular disulfide shuffling. Given little effects of the drugs on the band multiplicity, the drugs did not suppress abnormal shuffling of the disulfide bond within an apo-SOD1(G37R)^S-S^ molecule.

Shuffling of the disulfide bond in SOD1 proteins can be more directly examined with a disulfide mapping analysis using MALDI-TOF mass spectrometry followed by the limited tryptic digestion. In the absence of Gdn, tryptic digestion of apo-SOD1(G37R)^S-S^ produced mass peaks at *m/z* 4350 and 4507, which correspond to the peptides containing the canonical disulfide bond between Cys 57 and Cys 146 (Figure 6B, w/o Gdn). In contrast, several other mass peaks were emerged at *m/z* 4038 and 4607, when the apo-SOD1(G37R)^S-S^ sample agitated in the presence of 1 M Gdn was trypsinized (Figure 6B, no drugs). These mass peaks correspond to the peptides containing abnormal disulfide bonds between Cys 6 and Cys 57 (*m/z* 4038) and between Cys 111 and Cys 146 (*m/z* 4607), consistent with the shuffling of the disulfide bond. These four mass peaks observed at *m/z* 4038, 4350, 4507, and 4607 disappeared upon treatment of the samples with DTT before the measurement (data not shown), supporting that the observed mass peaks represent the peptides crosslinked *via* an intramolecular disulfide bond(s). In the presence of the drugs, nonetheless, the mass peaks of those peptides containing abnormal disulfide bonds were still observed (Figure 6B), confirming that the drugs were unable to suppress the disulfide shuffling process within a SOD1 molecule.

The drugs we found here can almost completely suppress the formation of insoluble SOD1 aggregates cross-linked *via* disulfide bonds. The drugs were not able to inhibit the disulfide shuffling within a SOD1 molecule but could act on the soluble disulfide-shuffled SOD1 molecules to stop further aggregation into the insoluble species (Figure 6C). Soluble SOD1 oligomers have been shown to exhibit significant toxicities toward cultured cells (Luchinat et al., 2014; Proctor et al., 2016), while identities of the soluble oligomers in the previous studies would not match those of soluble disulfide-shuffled SOD1 in our current study. Actually, it remains obscure whether the misfolded SOD1 with an intramolecularly-shuffled disulfide bond is monomeric, dimeric, or oligomeric. Potential toxicities of the soluble SOD1 species with a shuffled disulfide bond will also need to be examined, and our drugs might aggravate but not ameliorate the disease symptoms by stabilizing the soluble misfolded SOD1. Nonetheless, insoluble S-S oligomers are also observed as pathological species in the ALS-model mice; therefore, the drugs modulating the pathway for the formation of S-S oligomers will give pharmacological insights into the pathogenesis of *SOD1*-ALS.

### Caveats for the development of pharmaceuticals for SOD1-ALS based upon the drugs found in this study

Among the drugs reported here, statins have been reported to associate with the occurrence of neuromuscular degenerative disease and an ALS-like syndrome, albeit controversial (Edwards et al., 2007; Sorensen and Lash, 2009; Zheng et al., 2013). Actually, prescription of statins for men and women in their 60 s dramatically increased from 1991 to 1998 (Magrini et al., 1997; Riahi et al., 2001), but no similar increase of ALS incidence was observed (Fang et al., 2009). Side effects of statin medications include muscle toxicity such as myopathy (Sathasivam and Lecky, 2008), and limited evidence for neurotoxic effects of statins has been reported (Kiortsis et al., 2007). Nonetheless, a strong association between statin medications and functional decline/muscle cramping in patients with ALS has been reported (Zinman et al., 2008). While it remains unknown if statins are beneficial for ALS patients with *SOD1* mutations, administration of the statin (simvastatin) to *SOD1*-ALS model mice expressing human SOD1 with G93A mutation (G93A mice) was found to accelerate the disease progression and shorten the survival (Su et al., 2016). Given that a high level of lipids in serum is also proposed to be neuroprotective in ALS cases (Dupuis et al., 2008; Yoshii et al., 2010), treatment of ALS patients with statins inhibiting the biosynthesis of cholesterol should be cautious.

In contrast, vitamin D has been proposed as a potential therapy in ALS (Gianforcaro and Hamadeh, 2014), and several mechanisms describing positive effects of vitamin D on ALS have been suggested (Gianforcaro and Hamadeh, 2014). Among those, supplementation of vitamin D has been found to decrease the levels of pro-inflammatory cytokines such as TNF-α and would thereby reduce neuroinflammation in ALS (Gianforcaro and Hamadeh, 2014). Also, vitamin D has been suggested to have roles in muscle cell proliferation and differentiation and thus improve musculoskeletal functions (Gianforcaro and Hamadeh, 2014); indeed, vitamin D_3_ supplementation to the ALS patients reduced their decline in the ALSFRS-R score (Karam et al., 2013), which is a functional rating scale, and levels of vitamin D (serum calcidiol) appear to correlate with the ALS prognosis (Camu et al., 2014). Furthermore, vitamin D receptor-knockout mice exhibited significant impairment of locomotor and muscular functions, supporting a role of vitamin D in the maintenance of motor neurons (Burne et al., 2005). Deficiency of vitamin D_3_ in G93A mice was found to improve early disease severity and delays disease onset but with decline in the motor performance (Solomon et al., 2011); instead, supplementation of high doses of vitamin D_3_ to G93A mice improved their paw grip endurance and motor performance but with limited effectiveness on the disease course (Gianforcaro and Hamadeh, 2012; Gianforcaro et al., 2013). It is thus interesting to test in the future if vitamin D and/or its derivatives interact with mutant SOD1 to suppress the misfolding process and reduce their neurotoxicity in model mice.

Miltefosine is an antimicrobial drug that exhibits activity against parasites, pathogenic bacteria, and fungi (Dorlo et al., 2012), while no reports have been published on its efficacy to ALS and other neurodegenerative diseases. As summarized in the PubChem compound database (http://pubchem.ncbi.nlm.nih.gov), miltefosine has been reported to show toxicity toward some of human cells such as cancer cells, macrophages, and erythrocytes; therefore, a potential toxicity of miltefosine against neurons should be evaluated.

While the drugs found here (statins, vitamin D derivatives, and miltefosine) cannot be directly used for clinical treatment of *SOD1*-ALS, those drugs are found to specifically interact with a misfolded conformation of mutant SOD1 and thereby suppress the formation of insoluble oligomers/aggregates *in vitro* (Figure 6C). More effective and safer compounds with the ability to suppress the aggregation of SOD1 could hence be developed based upon our drugs and also be useful in evaluating the toxicity of misfolded/aggregated SOD1 in cultured cells to eventually modulate the disease course of *SOD1*-ALS cases.

## Author contributions

YF directed the project, analyzed the data and wrote the manuscript. IA, KT, and ET performed experiments and analyzed the data. AM and SA performed DSC experiments and analyzed the data. All authors reviewed the manuscript.

## Funding

This work was supported by Grants-in-Aid 16H04768 for Scientific Research (B) (to YF), 15H01566 for Scientific Research on Innovative Areas (to YF), 15K14480 for Challenging Exploratory Research (to YF), and 15H06588 for Young Scientists (Start-up) (to ET) from the Ministry of Education, Culture, Sports, Science, and Technology of Japan and also in part by Cooperative Research in Joint Studies at Institute for Molecular Science, Japan.

### Conflict of interest statement

The authors declare that the research was conducted in the absence of any commercial or financial relationships that could be construed as a potential conflict of interest.

## References

[B1] Abel O. Powell J. F. Andersen P. M. Al-Chalabi A. (2012). ALSoD: a user-friendly online bioinformatics tool for amyotrophic lateral sclerosis genetics. Hum. Mutat. 33, 1345–1351. 10.1002/humu.22157 22753137

[B2] Andersen P. M. Al-Chalabi A. (2011). Clinical genetics of amyotrophic lateral sclerosis: what do we really know? Nat. Rev. Neurol. 7, 603–615. 10.1038/nrneurol.2011.150 21989245

[B3] Antonyuk S. Strange R. W. Hasnain S. S. (2010). Structural discovery of small molecule binding sites in Cu-Zn human superoxide dismutase familial amyotrophic lateral sclerosis mutants provides insights for lead optimization. J. Med. Chem. 53, 1402–1406. 10.1021/jm9017948 20067275

[B4] Auclair J. R. Boggio K. J. Petsko G. A. Ringe D. Agar J. N. (2010). Strategies for stabilizing superoxide dismutase (SOD1), the protein destabilized in the most common form of familial amyotrophic lateral sclerosis. Proc. Natl. Acad. Sci. U.S.A. 107, 21394–21399. 10.1073/pnas.1015463107 21098299 PMC3003092

[B5] Banci L. Bertini I. Blazevits O. Calderone V. Cantini F. Mao J.. (2012). Interaction of cisplatin with human superoxide dismutase. J. Am. Chem. Soc. 134, 7009–7014. 10.1021/ja211591n 22471402

[B6] Benmohamed R. Arvanites A. C. Kim J. Ferrante R. J. Silverman R. B. Morimoto R. I.. (2011). Identification of compounds protective against G93A-SOD1 toxicity for the treatment of amyotrophic lateral sclerosis. Amyotroph. Lateral Scler. 12, 87–96. 10.3109/17482968.2010.522586 21073276 PMC8131116

[B7] Bruijn L. I. Houseweart M. K. Kato S. Anderson K. L. Anderson S. D. Ohama E.. (1998). Aggregation and motor neuron toxicity of an ALS-linked SOD1 mutant independent from wild-type SOD1. Science 281, 1851–1854. 10.1126/science.281.5384.1851 9743498

[B8] Burne T. H. Mcgrath J. J. Eyles D. W. Mackay-Sim A. (2005). Behavioural characterization of vitamin D receptor knockout mice. Behav. Brain Res. 157, 299–308. 10.1016/j.bbr.2004.07.008 15639181

[B9] Camu W. Tremblier B. Plassot C. Alphandery S. Salsac C. Pageot N.. (2014). Vitamin D confers protection to motoneurons and is a prognostic factor of amyotrophic lateral sclerosis. Neurobiol. Aging 35, 1198–1205. 10.1016/j.neurobiolaging.2013.11.005 24378089

[B10] Chattopadhyay M. Durazo A. Sohn S. H. Strong C. D. Gralla E. B. Whitelegge J. P.. (2008). Initiation and elongation in fibrillation of ALS-linked superoxide dismutase. Proc. Natl. Acad. Sci. U.S.A. 105, 18663–18668. 10.1073/pnas.0807058105 19022905 PMC2585484

[B11] Deng H. X. Shi Y. Furukawa Y. Zhai H. Fu R. Liu E.. (2006). Conversion to the amyotrophic lateral sclerosis phenotype is associated with intermolecular linked insoluble aggregates of SOD1 in mitochondria. Proc. Natl. Acad. Sci. U.S.A. 103, 7142–7147. 10.1073/pnas.0602046103 16636275 PMC1447523

[B12] Dorlo T. P. Balasegaram M. Beijnen J. H. De Vries P. J. (2012). Miltefosine: a review of its pharmacology and therapeutic efficacy in the treatment of leishmaniasis. J. Antimicrob. Chemother. 67, 2576–2597. 10.1093/jac/dks275 22833634

[B13] Dupuis L. Corcia P. Fergani A. Gonzalez De Aguilar J. L. Bonnefont-Rousselot D. Bittar R.. (2008). Dyslipidemia is a protective factor in amyotrophic lateral sclerosis. Neurology 70, 1004–1009. 10.1212/01.wnl.0000285080.70324.27 18199832

[B14] Edwards I. R. Star K. Kiuru A. (2007). Statins, neuromuscular degenerative disease and an amyotrophic lateral sclerosis-like syndrome: an analysis of individual case safety reports from vigibase. Drug Saf. 30, 515–525. 10.2165/00002018-200730060-00005 17536877

[B15] Fang F. Valdimarsdóttir U. Bellocco R. Ronnevi L. O. Sparén P. Fall K.. (2009). Amyotrophic lateral sclerosis in Sweden, 1991-2005. Arch. Neurol. 66, 515–519. 10.1001/archneurol.2009.13 19364937

[B16] Furukawa Y. (2012). Protein aggregates in pathological inclusions of amyotrophic lateral sclerosis, in Amyotrophic Lateral Sclerosis, ed Maurer M. H. (Rijeka: InTech), 335–356.

[B17] Furukawa Y. Anzai I. Akiyama S. Imai M. Cruz F. J. Saio T.. (2016). Conformational disorder of the most immature Cu, Zn-superoxide dismutase leading to amyotrophic lateral sclerosis. J. Biol. Chem. 291, 4144–4155. 10.1074/jbc.M115.683763 26694608 PMC4759189

[B18] Furukawa Y. Fu R. Deng H. X. Siddique T. O'Halloran T. V. (2006). Disulfide cross-linked protein represents a significant fraction of ALS-associated Cu, Zn-superoxide dismutase aggregates in spinal cords of model mice. Proc. Natl. Acad. Sci. U.S.A. 103, 7148–7153. 10.1073/pnas.0602048103 16636274 PMC1447524

[B19] Furukawa Y. Kaneko K. Yamanaka K. O'Halloran T. V. Nukina N. (2008). Complete loss of post-translational modifications triggers fibrillar aggregation of SOD1 in familial form of ALS. J. Biol. Chem. 283, 24167–24176. 10.1074/jbc.M802083200 18552350 PMC3259764

[B20] Furukawa Y. O'Halloran T. V. (2005). Amyotrophic lateral sclerosis mutations have the greatest destabilizing effect on the apo, reduced form of SOD1, leading to unfolding and oxidative aggregation. J. Biol. Chem. 280, 17266–17274. 10.1074/jbc.M500482200 15691826

[B21] Furukawa Y. Torres A. S. O'Halloran T. V. (2004). Oxygen-induced maturation of SOD1: a key role for disulfide formation by the copper chaperone CCS. EMBO J. 23, 2872–2881. 10.1038/sj.emboj.7600276 15215895 PMC1150991

[B22] Genç B. Özdinler P. H. (2014). Moving forward in clinical trials for ALS: motor neurons lead the way please. Drug Discov. Today 19, 441–449. 10.1016/j.drudis.2013.10.014 24171950 PMC4000750

[B23] Gianforcaro A. Hamadeh M. J. (2012). Dietary vitamin D3 supplementation at 10x the adequate intake improves functional capacity in the G93A transgenic mouse model of ALS, a pilot study. CNS Neurosci. Ther. 18, 547–557. 10.1111/j.1755-5949.2012.00316.x 22591278 PMC6493637

[B24] Gianforcaro A. Hamadeh M. J. (2014). Vitamin D as a potential therapy in amyotrophic lateral sclerosis. CNS Neurosci. Ther. 20, 101–111. 10.1111/cns.12204 24428861 PMC6493003

[B25] Gianforcaro A. Solomon J. A. Hamadeh M. J. (2013). Vitamin D(3) at 50x AI attenuates the decline in paw grip endurance, but not disease outcomes, in the G93A mouse model of ALS, and is toxic in females. PLoS ONE 8:e30243 10.1371/journal.pone.0030243 23405058 PMC3566148

[B26] Karam C. Barrett M. J. Imperato T. Macgowan D. J. Scelsa S. (2013). Vitamin D deficiency and its supplementation in patients with amyotrophic lateral sclerosis. J. Clin. Neurosci. 20, 1550–1553. 10.1016/j.jocn.2013.01.011 23815870

[B27] Kiortsis D. N. Filippatos T. D. Mikhailidis D. P. Elisaf M. S. Liberopoulos E. N. (2007). Statin-associated adverse effects beyond muscle and liver toxicity. Atherosclerosis 195, 7–16. 10.1016/j.atherosclerosis.2006.10.001 17094994

[B28] Luchinat E. Barbieri L. Rubino J. T. Kozyreva T. Cantini F. Banci L. (2014). In-cell NMR reveals potential precursor of toxic species from SOD1 fALS mutants. Nat. Commun. 5, 5502. 10.1038/ncomms6502 25429517

[B29] Magrini N. Einarson T. Vaccheri A. Mcmanus P. Montanaro N. Bergman U. (1997). Use of lipid-lowering drugs from 1990 to 1994: an international comparison among Australia, Finland, Italy (Emilia Romagna Region), Norway and Sweden. Eur. J. Clin. Pharmacol. 53, 185–189. 10.1007/s002280050360 9476029

[B30] McCord J. M. Fridovich I. (1969). Superoxide dismutase. An enzymic function for erythrocuprein (hemocuprein). J. Biol. Chem. 244, 6049–6055. 5389100

[B31] Mitomi Y. Nomura T. Kurosawa M. Nukina N. Furukawa Y. (2012). Post-aggregation oxidation of mutant huntingtin controls the interactions between aggregates. J. Biol. Chem. 287, 34764–34775. 10.1074/jbc.M112.387035 22891249 PMC3464579

[B32] Nowak R. J. Cuny G. D. Choi S. Lansbury P. T. Ray S. S. (2010). Improving binding specificity of pharmacological chaperones that target mutant superoxide dismutase-1 linked to familial amyotrophic lateral sclerosis using computational methods. J. Med. Chem. 53, 2709–2718. 10.1021/jm901062p 20232802 PMC2881568

[B33] Proctor E. A. Fee L. Tao Y. Redler R. L. Fay J. M. Zhang Y.. (2016). Nonnative SOD1 trimer is toxic to motor neurons in a model of amyotrophic lateral sclerosis. Proc. Natl. Acad. Sci. U.S.A. 113, 614–619. 10.1073/pnas.1516725113 26719414 PMC4725519

[B34] Rakhit R. Crow J. P. Lepock J. R. Kondejewski L. H. Cashman N. R. Chakrabartty A. (2004). Monomeric Cu,Zn-superoxide dismutase is a common misfolding intermediate in the oxidation models of sporadic and familial amyotrophic lateral sclerosis. J. Biol. Chem. 279, 15499–15504. 10.1074/jbc.M313295200 14734542

[B35] Ray S. S. Nowak R. J. Brown R. H. Jr. Lansbury P. T. Jr. (2005). Small-molecule-mediated stabilization of familial amyotrophic lateral sclerosis-linked superoxide dismutase mutants against unfolding and aggregation. Proc. Natl. Acad. Sci. U.S.A. 102, 3639–3644. 10.1073/pnas.0408277102 15738401 PMC553303

[B36] Ray S. S. Nowak R. J. Strolovich K. Brown R. H. Jr. Walz T. Lansbury P. T. Jr. (2004). An intersubunit disulfide bond prevents *in vitro* aggregation of a superoxide dismutase-1 mutant linked to familial amyotrophic lateral sclerosis. Biochemistry 43, 4899–4905. 10.1021/bi030246r 15109247

[B37] Renton A. E. Chiò A. Traynor B. J. (2014). State of play in amyotrophic lateral sclerosis genetics. Nat. Neurosci. 17, 17–23. 10.1038/nn.3584 24369373 PMC4544832

[B38] Riahi S. Fonager K. Toft E. Hvilsted-Rasmussen L. Bendsen J. Paaske Johnsen S.. (2001). Use of lipid-lowering drugs during 1991-98 in Northern Jutland, Denmark. Br. J. Clin. Pharmacol. 52, 307–311. 10.1046/j.0306-5251.2001.01439.x 11560563 PMC2014543

[B39] Rodriguez J. A. Shaw B. F. Durazo A. Sohn S. H. Doucette P. A. Nersissian A. M.. (2005). Destabilization of apoprotein is insufficient to explain Cu,Zn-superoxide dismutase-linked ALS pathogenesis. Proc. Natl. Acad. Sci. U.S.A. 102, 10516–10521. 10.1073/pnas.0502515102 16020530 PMC1175580

[B40] Rosen D. R. Siddique T. Patterson D. Figlewicz D. A. Sapp P. Hentati A.. (1993). Mutations in Cu/Zn superoxide dismutase gene are associated with familial amyotrophic lateral sclerosis. Nature 362, 59–62. 10.1038/362059a0 8446170

[B41] Sathasivam S. Lecky B. (2008). Statin induced myopathy. BMJ 337:a2286. 10.1136/bmj.a2286 18988647

[B42] Solomon J. A. Gianforcaro A. Hamadeh M. J. (2011). Vitamin D3 deficiency differentially affects functional and disease outcomes in the G93A mouse model of amyotrophic lateral sclerosis. PLoS ONE 6:e29354. 10.1371/journal.pone.0029354 22216257 PMC3246470

[B43] Sørensen H. T. Lash T. L. (2009). Statins and amyotrophic lateral sclerosis–the level of evidence for an association. J. Intern. Med. 266, 520–526. 10.1111/j.1365-2796.2009.02173.x 19930099

[B44] Stevens J. C. Chia R. Hendriks W. T. Bros-Facer V. Van Minnen J. Martin J. E.. (2010). Modification of superoxide dismutase 1 (SOD1) properties by a GFP tag–implications for research into amyotrophic lateral sclerosis (ALS). PLoS ONE 5:e9541. 10.1371/journal.pone.0009541 20221404 PMC2833207

[B45] Su X. W. Nandar W. Neely E. B. Simmons Z. Connor J. R. (2016). Statins accelerate disease progression and shorten survival in SOD1 mice. Muscle Nerve 54, 284–291. 10.1002/mus.25048 26799243 PMC5848093

[B46] Toichi K. Yamanaka K. Furukawa Y. (2013). Disulfide scrambling describes the oligomer formation of superoxide dismutase (SOD1) proteins in the familial form of amyotrophic lateral sclerosis. J. Biol. Chem. 288, 4970–4980. 10.1074/jbc.M112.414235 23264618 PMC3576100

[B47] Wright G. S. Antonyuk S. V. Kershaw N. M. Strange R. W. Samar Hasnain S. (2013). Ligand binding and aggregation of pathogenic SOD1. Nat. Commun. 4, 1758. 10.1038/ncomms2750 23612299 PMC3644087

[B48] Yoshii Y. Hadano S. Otomo A. Kawabe K. Ikeda K. Iwasaki Y. (2010). Lower serum lipid levels are related to respiratory impairment in patients with ALS. Neurology 74, 2027–2028. 10.1212/WNL.0b013e3181e03bbe 20548050

[B49] Zheng Z. Sheng L. Shang H. (2013). Statins and amyotrophic lateral sclerosis: a systematic review and meta-analysis. Amyotroph. Lateral Scler. Frontotemporal Degener. 14, 241–245. 10.3109/21678421.2012.732078 23134508

[B50] Zinman L. Sadeghi R. Gawel M. Patton D. Kiss A. (2008). Are statin medications safe in patients with ALS? Amyotroph. Lateral Scler. 9, 223–228. 10.1080/17482960802031092 18608105

